# Comparative analysis of human capital management strategies in the context of digitalization of the national economy

**DOI:** 10.3389/fsoc.2023.1114301

**Published:** 2023-04-25

**Authors:** Liudmila I. Khoruzhy, Valery I. Khoruzhy, Petr F. Kubrushko, Oksana G. Karataeva, Ludmila A. Bitkova

**Affiliations:** ^1^Russian State Agrarian University—Moscow Timiryazev Agricultural Academy, Moscow, Russia; ^2^Financial University under the Government of the Russian Federation, Moscow, Russia

**Keywords:** human resource management, innovation, entrepreneurship, change strategy, talent management, profit strategy

## 1. Introduction

The Fourth Industrial Revolution profoundly changed social institutions, industries, individuals, and ultimately the current society. The potential to improve the quality of life and generate economic benefits is one of the major promises of the Fourth Industrial Revolution, and some of the most significant opportunities can be found in nanotechnology, quantum technology, and artificial intelligence (AI). At the same time, the development and deployment of technology raise a range of ethical and social issues globally. These include (1) potential labor market disruptions, (2) biases inherent in the technology itself, (2) the emergence of new moral dilemmas, (3) societies torn apart by falling trust, (4) growing inequality due to the digital divide, and (5) growing threats to data privacy, security, and reliability (Harney and Collings, 2021). Solving these issues will make it possible to build a sustainable economy, adapt and modernize management models, reduce material and social inequality, and take responsibility for leadership in new technologies based on values. It is high time for leaders around the world to collaborate in the development and use of technology for the benefit of humanity, jointly overcome uncertainties, and shape a future that reflects shared values and humanity.

Within the framework of the digital format of economic relations focused on the transformation of all spheres of life, the priority trajectories are the use of processing a large amount of information, the introduction of digital technologies, and a new approach to managing human capital, which together will improve the quality of products (services), the efficiency of economic systems and competitiveness countries in general.

Therefore, one of the factors for effective digital modernization of society, as well as any process of change, can be the stimulation of skills of current workforce. In this regard, the issue of human development in the context of labor productivity growth during digitalization is very relevant and in high demand for scholarship and practice. Currently, companies are facing a number of problems related to human capital and its efficiency (Cooke et al., 2021; Zubak et al., 2021; Adisa et al., 2022). Dangerous trends are global changes in technology, demography, social instability, and climate problems, as well as human resources changes. The ecological situation, political, and interpersonal conflicts, and various forms of epidemics have an extremely detrimental effect on human resources, destroying sources of income, reducing the quality of food, and causing interruptions in the provision of basic medical and educational services (Boxall and Purcell, 2016; Farndale et al., 2020; Potgieter and Ferreira, 2022). These effects will manifest themselves over the course of many people's lives and are likely to reduce their productivity, so investing in human capital cannot be neglected.

The Human Capital Index (2020) shows that the productivity of almost 60% of children will be half of what they could achieve; at the same time, there are many positive examples of human resource transformation, for example, in Singapore, the Republic of Korea, and Ireland (Imran et al., 2020). This fact may indicate a crisis in the skills of current workforce and have a negative impact on economic growth in the future. The analysis of human resources is becoming a necessity since strategic personnel management is an important element and helps organizations to explore every side of HRM (Human Resources Management) indicators, according to Harney and Collings (2021).

Gallardo-Gallardo, Tunnissen, and Skullion believe that the COVID-19 pandemic has created a complex non-linear environment for HR professionals and practicing managers who are looking for original solutions to ensure that employees cope during a crisis and in a “new norm” to ensure continuity (Gallardo-Gallardo et al., 2020). Building on ongoing efforts to reconfigure HR in preparation for the “new normal” where employees must work from home (WFH) and perform tasks away from the workplace (WFW), one can draw on dynamic capability theory (DCT) to explain the adverse effects COVID-19 pandemic for operations, business models, and HR. Human capital directly affects the efficiency of organizations, and personnel management strategies will allow one to more thoroughly work out all aspects of intercommunications, as well as synthesize the areas of personnel management with the necessary results (Fahim, 2018; Armstrong and Brown, 2019; Mendy, 2022). It is extremely important to strategize further development, correctly prioritize, and identify the best tactics since the management of human resources is successful with the help of employees of a particular organization and the general direction of optimization.

Thus, the management and control of human resources are one of the priority areas for the digital transformation of society. The purpose of the study is to conduct a comparative analysis of human capital management strategies in the context of the digitalization of the national economy.

The study specifies the number of conceptual provisions of the “human capital” phenomenon, as well as the polyparadigmatic nature of the study of management strategies. First, we concretize the constituent postulates of human potential management strategies in the context of digitalization; second, they reveal the interdisciplinarity of this phenomenon. We have developed and tested a methodology that would perform complex modular measurements of human resources, and synthesize and evaluate the impact of micro-, meso-, and macro-factors on the economic performance of the digital environment.

The reorganization of the labor supply market and the development of digital platforms are transforming employment mechanisms, in connection with which we would like to highlight further the management strategies of the main human capital.

## 2. Materials and methods

The research aim is to compare human capital management strategies in the context of the digitalization of the national economy.

The research specifies several conceptual provisions of the phenomenon of “human capital” and the polyparadigmatic nature of the study of management strategies. Polyparadigmality implies the possibility of studying the management of human capital from different points due to the lack of a single theoretical framework.

The polyparadigmatic aspects of the problem converge at the point of a multidimensional typology of resources (HCR–human capital resource): (1) management and organization theory, (2) institutional economics, (3) market and firm theory, (4) behavioral dogmas of the organization, (5) various applied methodologies and methodologies for measuring and systematizing HCR, (6) business process human resources, and (7) strategic and collective human capital. When using the polyparadigm nature of the study of management research, it is important to consider the many paradigms of management research. The proposed study analyzes the polyparadigm of strategies (namely, profitability, liquidation, entrepreneurial, anti-crisis, and dynamic growth).

Table 1 presents the humanization of economic growth in the global economy of the world's leading economies: Germany, Russia, the USA, and Japan; big data is presented based on digital modeling.

**Table 1:** Key indicators of human capital development in the context of digitalization of several national economies.

**Country**	**2020**					**2021**					**2022**
	**PI**	**IEF**	**HIC**	**GDP_phw_**	**LDGOS**	**PI**	**IEF**	**HIC**	**GDP_phw_**	**LDGOS**	**IEF**
Germany	80.9	73.5	7.9	67.71	93.1	80.6	72.5	6.2	68.3	73.5	76.1
Russia	58.7	61	10	27.68	no data	59.3	61.5	8.4	no data	81.8	56.1
USA	77.5	76.6	12.1	73.37	no data	77.1	74.8	10.6	74.1	94.7	72.1
Japan	77.6	73.3	8.1	48.13	95.1	77.7	74.1	7.9	45.52	90.6	69.9

LDGOS–is the level of development of the government's online service, rated in points (1–100) and calculated by WIPO; data from some countries for 2020 and 2022 were not taken into account due to lack of data (no data). The highest score in 2021 was occupied by the USA (94.7), and the lowest–by Germany (73.5). One can assume that the digitalization of government's online services actively influences the development of human capital and strategies for its management.

PI–is the prosperity index, rated in points (1–100) and calculated by the Legatum Institute Foundation; data is contained in The Legatum Prosperity Index™ 2019: Creating the Pathways from Poverty to Prosperity Report and reflects the achievements of the countries of the world in terms of their wellbeing and prosperity. PI practically did not change for the considered countries in 2020–2021. This index can also be taken into account in modern personnel strategies for managing human capital.

IEF–is the index of economic freedom, rated in points (1–100) and calculated by the Heritage Foundation; data is contained in Country Rankings of Economic Freedom 2020 and reflects the rule of law, the degree of bureaucratization of public administration, the effectiveness of regulation, and the openness of markets. In 2022, Germany had the highest IEF (76.1), and Russia–the lowest (56.1). IEF can also be considered in new personnel management strategies.

HIC–is the human inequality coefficient calculated by the United Nations (UNDP); data is contained in the Human Development Report 2019. It reflects the degree of social justice in education, employment, career development, performance, and pay (in %). In 2021, the USA had the highest HIC (10.6), and Germany–the lowest (6.2). The index can be integrated into modern strategies.

GDP_phw_-is the productivity per capita (GDP per hour worked) per hour (USD). It is calculated by the OECD and shows the cost of goods produced by one worker per 1 h of work. This index can be taken into account in digital HR management strategies. Unfortunately, there is no information about GDP_phw_ in Russia today; in the USA, the index is 71.1 USD.

The novelty of the proposed research is characterized, first, by the specification of the conceptual dogmas of human capital administration in the context of digitalization and, second, by the assumption of the interdisciplinarity of the digitalization phenomenon. We tested the skills of current workforce methodology, which is based on the calculations of the World Economic Forum (the Global Competitiveness Report) and reflects the level of education and training, labor force qualifications, and digital skills of the population.

## 3. Main strategies for the management of human capital in the context of digitalization of the national economy

Strategic management originated in the early 80s of the 20th century at the enterprises of Western Europe. Strategic management of skills of current workforce involves managing the formation of competitive labor potential, taking into account current and future transformations of the external and internal environment, which allows the organization to survive, develop, and achieve short-term and long-term goals. The reorganization of the labor supply market and the development of digital platforms are transforming employment mechanisms; in this connection, further, we will highlight the main current personnel strategies.

### 3.1. Human resource management profitability strategy

The main strategy is the concept of profitability of human capital management in the context of digitalization of economic systems (Hamburg, 2019; Zubak et al., 2021). This strategy involves maintaining the level of profits while reducing financial costs, does not involve hiring new employees, and uses a digital network of inter-rank management. The polyparadigm aspects of profitability strategy (GDP_phw_; PI) are related to the behavioral dogmas of the organization and methodologies for measuring and systematizing HCR. For example, when calculating the profitability of a business and the cost of an hour using modeling methods for 50 production employees who directly provide services to customers (for example, consultants, engineers, and auditors), the full salary per hour will be 330 rubles, and the profitability of the business will be 59%. In the considered strategy, recruitment is carried out according to strict criteria; wages are calculated depending on the length of service.

The advantage of the strategy of profitability of human resource management in the context of the digitalization of national economic systems is the focus on flexible management of personnel training. The classical methods of managing human potential based on the development of skills no longer meet the requirements of the modern labor market, according to the concept developed by I. Hamburg (2019). The demanding challenge of the digital age is that technology is rapidly changing, and the evolution of human resources determines industry competitiveness, so corporate learning based on continuous learning in the profitability strategy is activated.

In addition, corporate learning extends beyond a single company, with massive open online courses and social learning becoming especially popular. These trends are producing new types of corporate training and co-working spaces.

### 3.2. Liquidation strategy of human resources management

The next direction in the control of human capital in the context of the digitalization of national economies is the liquidation concept (Cooke et al., 2021). This strategy is focused on reducing the activity of activities associated with the factors reducing company profits. The polyparadigm aspects of liquidation strategy (GDP_phw_; IEF) converge on a multidimensional typology of HCR resources: management and organization theory, behavioral dogmas of the organization, HCR systematization, and strategic and collective human capital. For example, when transferring employees to a remote work format, eliminating rental fees, and introducing automation everywhere, business profits can increase up to 10%. Financial rewards in an organization using a liquidation strategy are gradually increased based on merit, and staff skills are constantly improved as needed. The liquidation strategy in the context of the digitalization of economies in the field of human capital management includes the automation of the functions of the personnel department, which is an important advantage of the concept. Automation includes, on the one hand, the introduction of digital workplaces and, on the other hand, technologies that change the methods of work and interaction between employees (Harney and Collings, 2021). Currently, there are many HR products, and solutions on the market focused on mobile applications, cloud services, and artificial intelligence. The liquidation concept, in contrast to the profitability strategy, adapts artificial intelligence based on the reflection of the results and methods for achieving the goal and minimizes personnel risks.

### 3.3. Entrepreneurial strategy of human capital management

The next relevant concept of human resources management in the context of the digitalization of national economic systems is an entrepreneurial strategy (Malik and Sanders, 2021). The polyparadigm aspects of entrepreneurial strategy (GDP_phw_; IEF; PI) converge on the management, the theory of behavioral dogmas of the organization, and strategic and collective human capital. At present, one can state the formation of an innovative direction in management based on the management of human potential, a dynamic network organizational structure focused on the cultivation and management of self-managed working groups. The spread of remote work contributes to the acquisition of skills for online management of virtual departments and well-coordinated work in a multicultural environment, which also implements an entrepreneurial strategy. The advantage of this strategy is that it is implemented in conditions of a high degree of financial risk and limited resources and involves mobile decision-making. Personnel awards that meet personal needs are awarded on a competitive basis, with an emphasis on entrepreneurial strategy and teamwork. In contrast to the liquidation strategy, the entrepreneurial concept is focused on the use of digital platforms that allow economic activity to be regulated in such a way that most of the duties performed by full-time employees can be transferred to a private company.

### 3.4. Anti-crisis strategy of human resources management

The anti-crisis strategy for the transformation of human resources management in the context of the digitalization of national economies is aimed at preventing the bankruptcy of an organization (Mendy, 2022). The polyparadigm aspects of anti-crisis strategy (PI; IEF) converge at the point of measurement and systematization of HCR and the business process of human resources. One should note that bankruptcy can be not only organizational and financial. The bankruptcy of an organization also implies the default of humanism and the reduction of people to the level of production values. The anti-crisis strategy implies a sharp reduction in costs, as well as personnel to stabilize the overall situation; therefore, external and internal personnel support reforms are being carried out. Rewards are closely related to performance, but moral encouragement is possible. The anti-crisis strategy for the transformation of human resources management in the context of the digitalization of the national economy is focused on talent management (Gallardo-Gallardo et al., 2020; Trébucq and Belghit, 2021). The innovation lies in the fact that HR managers strive to retain their best employees and are focused on the development of less ambitious employees. There is a growing interest in employees with high but unrealized potential. A career matrix is being developed based on corrective actions. The digital system ranks individual employee courses and stores “digital tracks” that can be monitored. This information saves time and has a positive impact on the productivity of managers.

### 3.5. Dynamic growth strategy for human resources management

In the human resource management system, the concept in the context of the digitization of national economies is focused on low risks and sustainable growth, and long-term planning is the dynamic growth strategy of Knice et al. (2018). Polyparadigmatic aspects of the strategy (IEF; PI; HIC) of dynamic growth converge at the point of a multidimensional typology of resources of the institutional economy, the theory of markets and the organization, business processes of the human reserve, and strategic and collective human capital. The advantage of the strategy is that the results of the employee are periodically monitored (certified) for compliance with the general goals of the company. It is assumed that the staff is attached to a specific position with a set of functional tasks, but in case of difficulties, the interchangeability of structures is also possible. Material and non-material remuneration is awarded to an employee in accordance with clear criteria. When implementing a dynamic growth strategy, much attention is paid to digital communications (social networks, gadgets), in contrast to the strategies discussed above. HR departments use social media not only for hiring but also to attract employees, as well as to send out industry news.

Any strategy for the development and control of human potential accumulates the conditions and safety of work, methodological aspects of conflict resolution, methods for assessing and adapting personnel, a code of corporate ethics, a personnel reserve, a system of incentives and remuneration, as well as a development concept. The modernization of human potential in the context of the digitalization of the economy should be based on a competency-based approach while taking into account the new conditions; digital competencies will be a priority.

## 4. Conclusion

As a result, we provide a detailed outlook on the cumulative challenges associated with intra-entrepreneurship, the sustainability of HRM management, and the digital transformation facing modern organizations. The researchers considered a sustainable approach to personnel management, which focuses on the synthesis of internal and external factors in the context of achieving positive results not only for a particular institution but also for the socio-economic segment and the environment. There is a strong necessity to examine cases related to the role of HRM in creating a corporate image and optimal development of institutions, as well as in increasing employee engagement, satisfaction, productivity, and wellbeing. The study discusses the new opportunities provided by digitalization and mobile communications in the field of intellectual capital, which make employees the focus of an organization to create sustainable, highly competitive advantages, which is the practical significance of the study.

## Author contributions

Research design: LK. Research: VK, PK, and LB. Literature review: PK and LB. Writing: LK and VK. All authors contributed to the article and approved the submitted version.
